# Extracellular Vesicles Isolated from Malignant Mesothelioma Cancer-Associated Fibroblasts Induce Pro-Oncogenic Changes in Healthy Mesothelial Cells

**DOI:** 10.3390/ijms232012469

**Published:** 2022-10-18

**Authors:** Tatyana Chernova, Stefano Grosso, Xiao-Ming Sun, Angela Rubio Tenor, Joaquin Zacarias Cabeza, Andrew Craxton, Emily L. Self, Apostolos Nakas, Kelvin Cain, Marion MacFarlane, Anne E. Willis

**Affiliations:** 1 MRC Toxicology Unit, University of Cambridge, Tennis Court Rd., Cambridge CB2 1QR, UK; 2 UHL NHS Trust, Glenfield Hospital, Leicester LE3 9QP, UK

**Keywords:** cancer, asbestos, mesothelioma, fibroblasts, extracellular vesicles, tumour microenvironment

## Abstract

Malignant mesothelioma is an aggressive tumour of the pleura (MPM) or peritoneum with a clinical presentation at an advanced stage of the disease. Current therapies only marginally improve survival and there is an urgent need to identify new treatments. Carcinoma-associated fibroblasts (CAFs) represent the main component of a vast stroma within MPM and play an important role in the tumour microenvironment. The influence of CAFs on cancer progression, aggressiveness and metastasis is well understood; however, the role of CAF-derived extracellular vesicles (CAF-EVs) in the promotion of tumour development and invasiveness is underexplored. We purified CAF-EVs from MPM-associated cells and healthy dermal human fibroblasts and examined their effect on cell proliferation and motility. The data show that exposure of healthy mesothelial cells to EVs derived from CAFs, but not from normal dermal human fibroblasts (NDHF) resulted in activating pro-oncogenic signalling pathways and increased proliferation and motility. Consistent with its role in suppressing Yes-Associated Protein (YAP) activation (which in MPM is a result of Hippo pathway inactivation), treatment with Simvastatin ameliorated the pro-oncogenic effects instigated by CAF-EVs by mechanisms involving both a reduction in EV number and changes in EV cargo. Collectively, these data determine the significance of CAF-derived EVs in mesothelioma development and progression and suggest new targets in cancer therapy.

## 1. Introduction

Malignant mesothelioma is an aggressive, fatal tumour of the pleura (MPM) or peritoneum that is related to asbestos exposure and exhibits a very long latency period, with disease onset occurring after ~40 years [1]. To gain a mechanistic understanding of this devastating disease, multiple analyses have been performed on MPM tumours, and the data show that, for the most part, MPM is associated with genetic alterations that decrease tumour suppressor gene function with no oncogenic drivers identified [2,3,4,5,6]; importantly these data are replicated in animal studies [7]. In addition to genomic changes, it has been shown that the translatome is also dysregulated in MPM [8]. These combined studies [2,3,4,5,8] are leading to new therapeutic directions for MPM [6,8], but at present, the standard of care for MPM, chemotherapy based on a combination of pemetrexed and cisplatin or radical surgery, is only modestly effective at prolonging the life of patients [1,9]; although therapies that target immune system checkpoints do show promise [10,11]. 

An alternative way to examine therapeutic liabilities in MPM is to explore the relationship between mesothelial cells and carcinoma-associated fibroblasts (CAFs) [12]. Although several studies have identified changes in the expression and activity of defined cell signalling pathways in mesothelial and stromal cells [13,14], the relationship between different cell types in the process of tumourigenesis has not been studied in detail. This is important as MPM is a tumour with a substantive stromal component that includes a range of non-tumour cells [12]. CAFs represent the main component of tumour stroma with considerable infiltration in specimens from MPM patients [7,15]. Activation of pro-oncogenic signalling pathways is prominent in the stromal component, including CAFs [7]. Fibroblasts, when activated, play a central role in promoting migration and invasion of cancer cells, modulating chemoresistance and influencing cancer metabolism [16]. It has been shown that CAFs induce tumour proliferation and motility [17] and promote EMT in different types of cancer [18], favouring metastasis [19,20]. Emerging new therapies target CAFs with promising results in vivo [21,22]. This highlights the importance of pursuing in-depth analyses of the functionality of CAFs and their specific contribution to asbestos-induced carcinogenesis. 

Cell-to-cell communication by transfer of biological material, shedding and uptake of small vesicles is now considered a vital part of cell crosstalk and particular interest in the context of the tumour microenvironment [23,24]. Extracellular vesicles (EVs) are part of the cell secretome with versatile cargo that, upon uptake, can change a recipient cell’s molecular makeup and function [25]. These effects of tumour-derived EVs have been extensively studied [17,26,27,28] and have been reported to cause activation of fibroblasts towards a CAF phenotype in some cancers [29]. For example, CAF-derived EVs were shown to transfer lipids and proteins to cancer cells to support tumour growth [17], including gain-of-function p53 [30], and pleiotropically modulate cancer cell metabolism [27] in prostate and pancreatic cancer, respectively. 

In this study, we investigate cellular crosstalk between primary CAFs from MPM and mesothelial cells using EV release and uptake. We also demonstrate inhibition of the pro-oncogenic effects of CAF-EVs by Simvastatin, consistent with its role in suppressing Yes-Associated Protein (YAP) activation, which in mesothelioma results from an inactivation of the Hippo pathway [31,32]. 

## 2. Results

### 2.1. Primary CAFs Display an Activated Phenotype 

**Figure 1:**
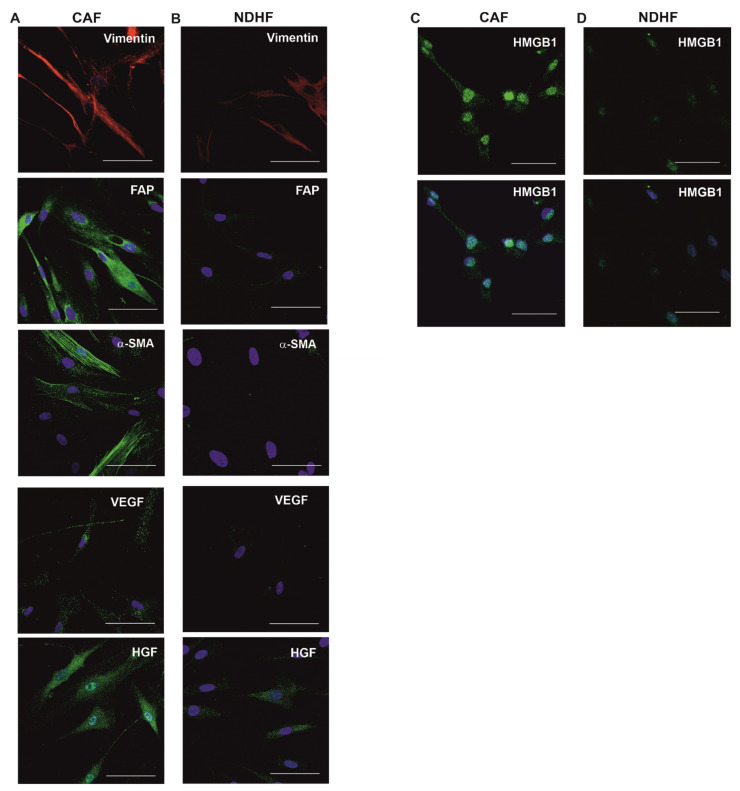
Activation profile of CAFs and NDHFs. CAFs (**A**) display a higher level of expression of the proteins associated with fibroblast activation compared to NDHFs (**B**). Vimentin (red); FAP, α-SMA, VEGF and HGF (all green), DAPI (blue). (**C**,**D**) Positive cytoplasmic staining for the DAMP protein, HMGB1, in CAFs (**C**) and negative staining in NDHFs (**D**); HMGB1 (green) upper panel, HMGB1 (green) and DAPI (blue) lower panel. Scale bars, 50 μm.

A panel of markers and proteins associated with fibroblast activation were examined in primary CAFs, isolated from resected mesothelioma tumour tissue [33] and in normal dermal human fibroblasts (NDHFs) (Figure 1A,B). As expected, both types of fibroblasts displayed expression of the mesenchymal cell marker Vimentin, but expression levels were higher in CAFs. The expression of Fibroblast Activation Protein (FAP), a member of the serine protease family, was detected in CAFs but not in NDHF, consistent with their activation status. CAFs expressed α-smooth-muscle actin, which also indicates the reactive state of the cells. The expression of the Hepatocyte Growth Factor (HGF) and Vascular Endothelial Growth Factor (VEGF), both proteins with pro-tumorigenic qualities [34,35], was markedly higher in CAFs compared to NDHFs. Immunostaining for the DAMP, high-mobility group protein B1 (HMGB1), revealed strong positive nuclear expression and, notably, protein cytoplasmic translocation in CAFs, indicative of the release of HMGB1 by these cells. In contrast, in NDHFs, HMGB1 expression was lower and limited to the nuclei (Figure 1C,D). Furthermore, CAFs were major contributors to the activation of canonical pro-oncogenic signalling pathways in the whole mesothelioma tumour tissue (Figure S1). Importantly, key oncogenic signalling pathways, including ERK1/2, mTOR, p38, MSK, STAT5, STAT3 and PDGF, were induced in CAFs and less so in the mesothelial cells from the same tumour.

### 2.2. Normal Untransformed Mesothelial Cells LP-9 Internalize CAF-Derived Extracellular Vesicles

**Figure 2:**
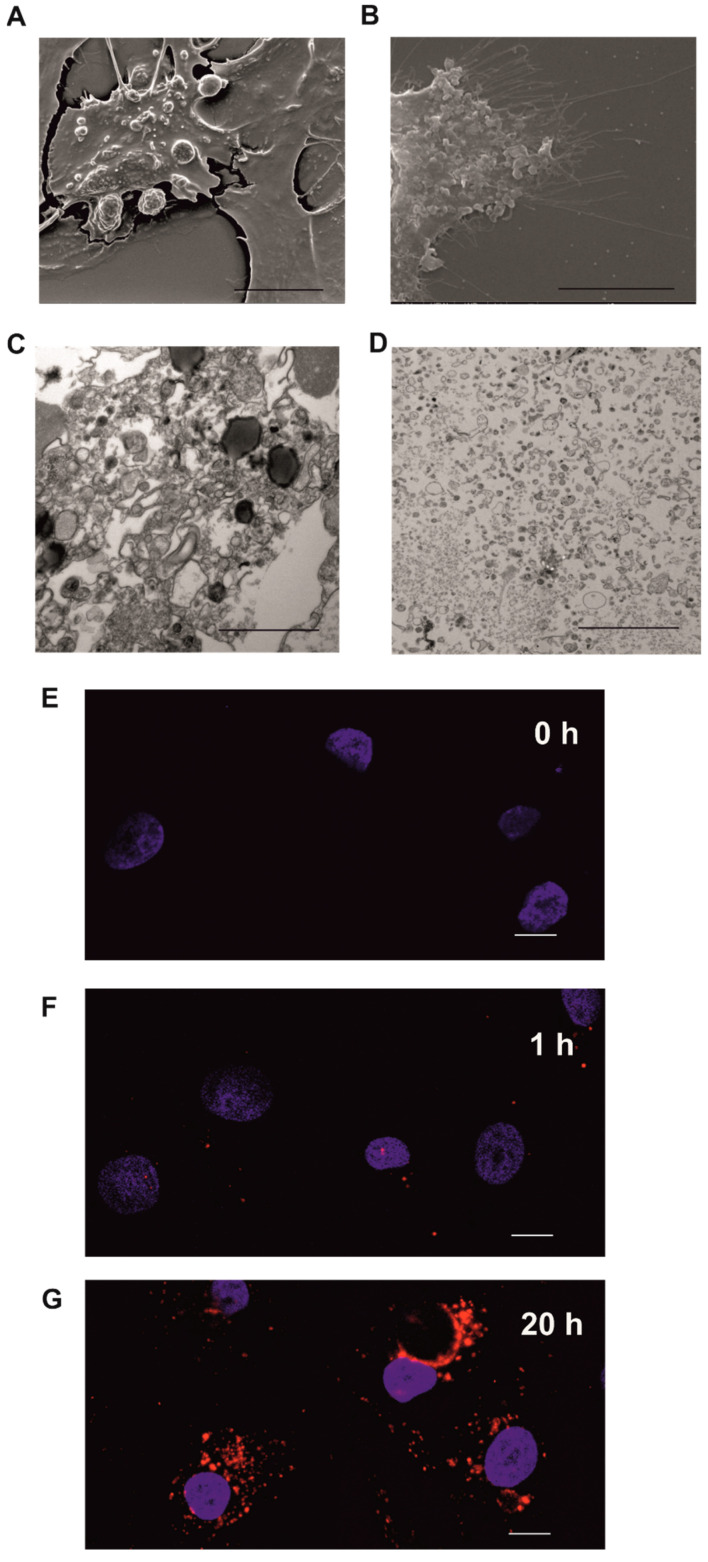
CAF-derived EVs are internalised by normal mesothelial cells. (**A**,**B**) SEM images of the surface of CAFs (**A**) and NDHFs (**B**). Scale bars, 10 μm. (**C**,**D**) TEM of EVs derived from CAFs (**C**) and NDHFs (**D**), pelleted by differential centrifugation. Scale bars, 10 μm. (**E**–**G**). Representative z-stack analysis of confocal images depicting internalisation of PKH26-labeled human CAF-derived EVs (red) by LP-9 cells detected by confocal microscopy at 0 h (**E**), at 1 h (**F**) and 20 h (**G**), DAPI (blue). Three independent experiments were performed with similar results. Scale bars, 10 μm.

EVs were isolated from conditioned media collected from fibroblast cultures. The morphological appearance of CAF- and NDHF-derived EVs was compared using electron microscopy. The population of NDHF-derived vesicles was more uniform when examined by both Scanning Electron Microscopy (SEM) of the fibroblast surface and Transmission Electron Microscopy (TEM) of the EV pellets isolated from CAF and NDHF cultures (Figure 2A–D). To visualise CAF-EV internalisation by the untransformed mesothelial cells LP-9, EVs were labelled with the fluorescent dye PKH-26 and then incubated with LP-9 cells. After 20 h incubation, more than 90% of LP-9 cells were PKH-26 signal positive, indicating EV transfer to recipient cells (Figure 2E–G).

### 2.3. CAF-Derived Vesicles Activate Pro-Oncogenic Signalling Pathways in Normal Mesothelial Cells 

**Figure 3:**
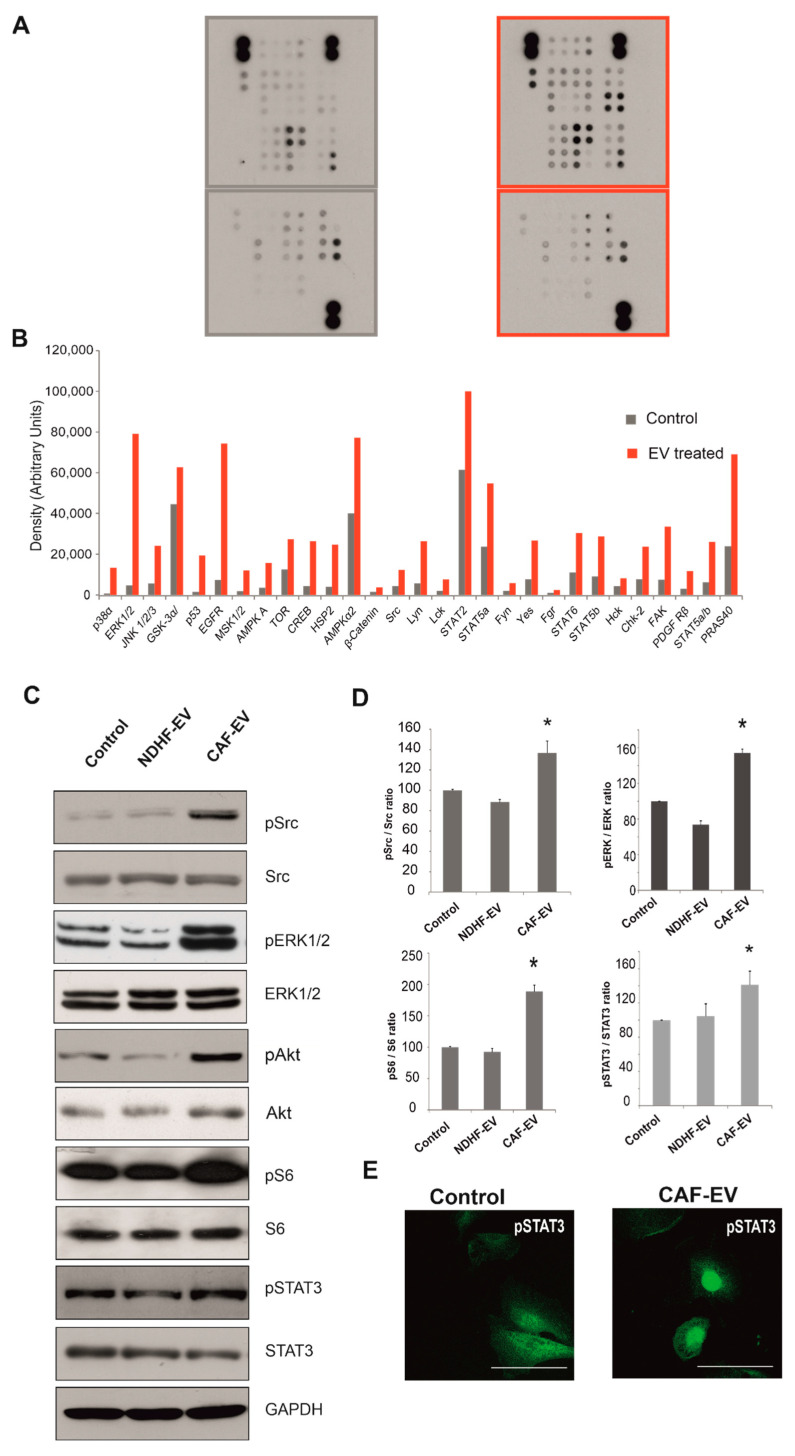
CAF-derived EVs induce pro-oncogenic signalling pathways in normal mesothelial cells. (**A**,**B**) The image shows the immunoblots of kinases in LP-9 cells treated with EVs derived from NDHFs (left) and CAFs (right, red) for 24 h. (**B**) Kinase activation status quantified by densitometry and showing induction of pro-oncogenic pathways in LP-9 cells in response to CAF-derived EVs. (**C**) LP-9 cells treated with CAF-EVs and NDHF-EVs for 24 h were analysed for phospho-Src (T416), phospho-ERK1/2 (T202/204), phospho-S6 (S235/236), and phospho-STAT3 (Y705) by western blotting and compared to untreated normal mesothelial cells (Control). Representative data from three experiments. (**D**) Quantification of phosphorylated and total protein ratios in LP-9 cells treated with CAF- EVs, NDHF- EVs or untreated (Control). Error bars show mean ± SD from three independent experiments. Statistical significance was estimated using a two-tailed Student’s *t*-test, * *p* < 0.05. (**E**). Representative image of nuclear translocation of phospho-STAT3 (Y705) protein (green) in LP-9 cells treated with CAF-EVs for 24 h. Scale bars, 50 μm.

The status of 64 kinases was profiled in LP-9 cells treated with CAF-derived EVs. This treatment markedly induced pro-oncogenic signalling pathways, including ERK1/2, Src Family Kinases (SFK), Akt, mTOR and STATs (Figure 3A,B). The results of kinome profiling were validated by immunoblotting (Figure 3C,D). Increased phosphorylation of several pro-oncogenic signalling pathways, including Src family kinases, ERK, Receptor Tyrosine Kinases (RTK) and STAT family transcription factors, including nuclear translocation of phospho-STAT3 (Figure 3E), were evident in CAF-EV treated LP-9 cells. By contrast, EVs derived from NDHFs did not affect the activation level of these proteins (Figure 3C,D). There was a marked increase in phosphorylation of S6 ribosomal protein, known to correlate with an increase in mRNA translation [36], as a result of CAF-EV but not NDHF treatment. These pathways were also activated when patient mesothelioma tissues were profiled (Figure S1) and the data are consistent with recent work that shows protein synthesis is activated in MPM [8].

### 2.4. CAF- but Not NDHF-Derived EVs Induce Proliferation in Normal Mesothelial Cells 

**Figure 4:**
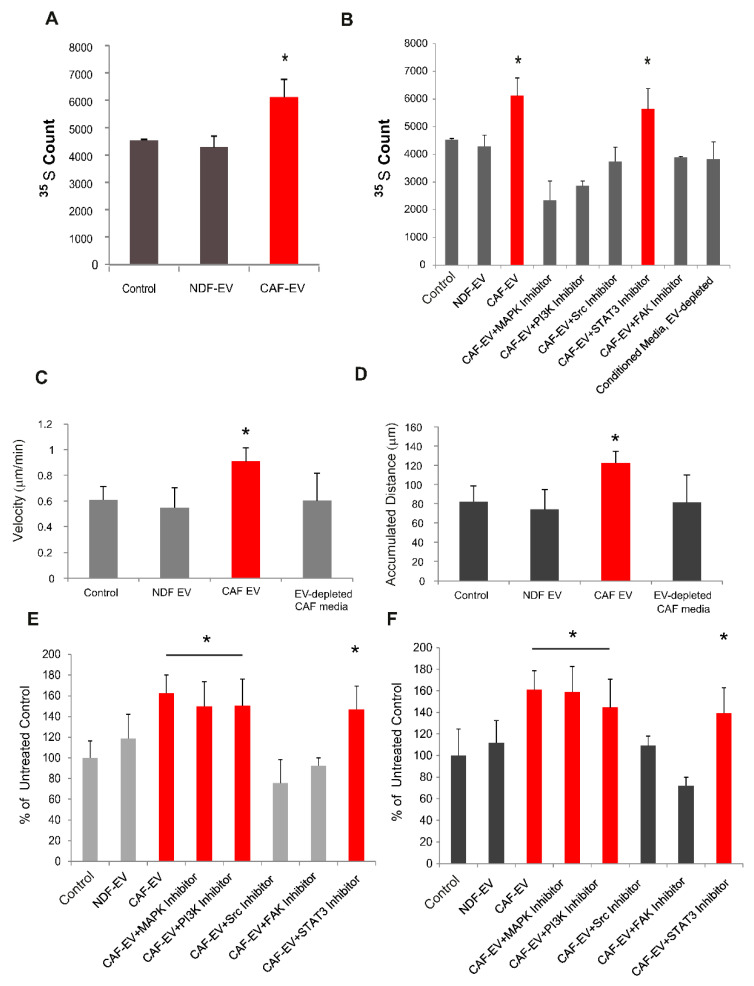
CAF-derived EVs increase translational activity and motility of mesothelial cells. (**A**) Met 35S incorporation in LP-9 cells treated with CAF- or NDHF-derived EVs for 24 h compared to untreated LP-9 cells (Control). (**B**) The translational activity of LP-9 cells treated with CAF-EVs in the presence or absence of inhibitors of MAPK, PI3K, Src, STAT3 and FAK. Induction of mRNA translation by CAF-EVs is MAPK- and PI3K-dependent. Velocity (**C**) and accumulated distance (**D**) of LP-9 cells treated with CAF- or NDHF-derived EVs for 24 h, compared to treatment with EV-depleted CAF-conditioned media or untreated control. Velocity (**E**) and accumulated distance (**F**) of LP-9 cells treated with CAF-EVs in the presence or absence of kinases inhibitors for 24 h expressed as a percentage of untreated control cells. In all panels, error bars show mean ± SD from three independent experiments with statistical significance estimated using the two-tailed Student’s *t*-test, * *p* < 0.05.

To examine how aberrant signalling exerted by EVs affected functional readouts, normal mesothelial LP-9 cells were treated with EVs derived from CAFs and NDHFs. Protein synthesis rates in LP-9 cells were assessed by ^35^S Met incorporation assay, and these were significantly increased by CAF-EV treatment (Figure 4A). In contrast, EVs derived from NDHFs did not affect mRNA translation in LP-9 cells (Figure 4A). The process of mRNA translation is downstream of the majority of proliferative signalling pathways, and the data show that the CAF-EV-induced increase in protein synthesis of LP-9 cells was MAPK-, PI3K- and Src Family Kinase (SFK)-dependent (Figure 4B); with the stimulatory effect of CAF-EVs abolished when LP-9 cells were co-treated with specific inhibitors of these pathways. By contrast, inhibition of the STAT3 pathway did not abolish mRNA translation activation (Figure 4B).

### 2.5. CAF-Derived EVs Increase Motility of Normal Mesothelial Cells

A change from stationary to motile state is characteristic of cell malignant transformation. To investigate if EVs affect the motility of normal mesothelial cells, LP-9 cells were treated with EVs for 24 h, and their motility was monitored over 16 h, between 8 and 24 h following treatment (Figure 4C,D). CAF- but not NDHF-derived EVs increased both the velocity and accumulated distance migrated by LP-9 cells. Consistent with their previously reported roles in cell migration-associated signalling pathways, the induction of cell motility was both Src- and FAK-dependent.

### 2.6. Simvastatin Reduces Pro-Oncogenic CAF-EV-Induced Signalling 

**Figure 5:**
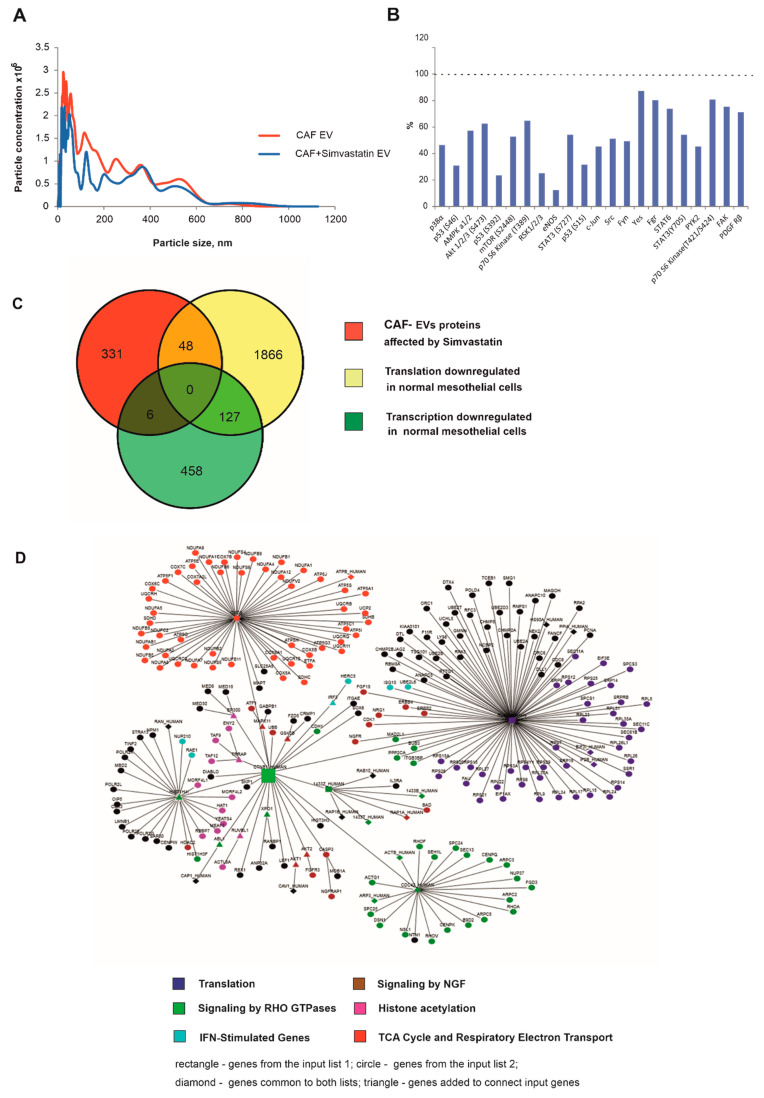
Simvastatin treatment reduces EVs production by CAFs and ameliorates activation of pro-oncogenic signalling pathways in LP-9 cells. (**A**) Representative graph of concentration and size distribution of EVs derived from the same number of untreated CAFs and CAFs treated with 500 nM Simvastatin for 24 h, obtained by nanoparticle tracking analysis (NTA). (**B**) Kinome profiling analysis of LP-9 cells treated with EVs derived from CAFs. Kinase activation in LP-9 cells incubated with EVs derived from Simvastatin-treated CAFs expressed as a percentage of that detected in LP-9 cells incubated with an equal amount of EVs derived from untreated CAFs (set to 100%). (**C**) Venn diagram showing overlap of the genes transcriptionally or translationally downregulated (*p* < 0.05) in normal mesothelial cells with the genes encoding proteins affected by Simvastatin treatment. (**D**) The top genes functionally related to the two lists of genes (1st—affected by Simvastatin treatment, 2nd—translationally downregulated in normal mesothelial cells compared to malignant cells) with *p* < 0.00096, Catenin beta 1 (CTNNB1), forms a network related to translation, mitochondrial function, chromatin remodelling, signalling by RHO GTPases, NGF and Interferon (rectangle—the gene from the input list 1; circle—the gene from the input list 2; diamond—the gene common to both lists; triangle—genes added to connect input genes).

It has previously been shown that the statin, Simvastatin, mediates inhibition of exosome synthesis, localisation and secretion via multicomponent interventions [37]. We, therefore, used Simvastatin as a tool to explore the effects of statins on CAF-EVs and their cargo, focusing on EV-related crosstalk between CAFs and mesothelial cells. CAFs were treated with 500 nM Simvastatin for the duration of EV collection (24 h), with the same number of untreated CAFs used to collect EVs. The EVs collected from Simvastatin-treated and untreated fibroblasts were compared in terms of their ability to induce pro-oncogenic signalling in normal mesothelial cells. Measuring the concentration of EVs by a nanoparticle tracking analyser revealed a significant reduction in total EVs released by Simvastatin-treated CAFs; when total EVs released by matched untreated CAFs was set to 100%, total EVs released by Simvastatin-treated CAFs were reduced to 48.7 ± 13.4% (Mean ± SD, *n* = 3 mesothelioma patients; representative graph Figure 5A). Treatment with Simvastatin ameliorated the pro-oncogenic effects of CAFs on normal mesothelial cells. When EVs collected from Simvastatin-treated CAFs were used to treat LP-9 cells, the activation of signalling pathways was less prominent compared to that induced by EVs from untreated CAFs (Figure 5B). This effect of Simvastatin could be due to a reduction of EV release by CAFs, as well as a change in EV cargo content as determined by Mass Spectrometry (Figure S2A). The change in CAF-EVs cargo content was analysed using an analytical web portal for high-throughput cell biology BioProfiling.de: http://www.bioprofiling.de/gene_list (accessed on 30 May 2022) [38]. This revealed that the proteins whose content in CAF EVs changed after Simvastatin treatment form a network of pathways related to metabolism, cell cycle, growth factors, integrins and lipid transport (Figure S2C). Translational and transcriptional analysis of primary normal mesothelial cells and primary MPM cells in our previous work has identified those genes that are statistically significantly downregulated in normal cells compared to malignant MPM [33]. Further analysis of Mass Spectrometry results, along with the comparison of the transcriptome and translatome of primary normal and malignant mesothelial cells, was conducted to see how EVs cargo relates to those proteins in primary normal mesothelial cells (Figure 5C). It was revealed that the proteins differentially present in EVs were downregulated in normal compared to malignant mesothelial cells, i.e., they are not actively translated in normal mesothelial cells (Figure 5C). Thus, delivery of these proteins to normal cells by EVs would cause a marked change in cellular makeup. A strong functional link (*p* < 0.001) was determined between the list of genes with downregulated translation in normal mesothelial cells and the list of proteins differentially present in treated and untreated CAF-EVs. Those genes functionally linked include those related to the Wnt/beta-Catenin and the NF-kappa B signalling pathways, integrins, genes involved in the regulation of cell growth, translation, protein degradation, DNA repair and replication, mitochondrial function and regulation of the cell cycle (Tables S1 and S2). The top gene functionally linked to both lists with *p* < 0.00096, Catenin beta 1 (CTNNB1), is part of a network related to mRNA translation, mitochondrial function, chromatin remodelling, signalling by RHO GTPases, NGF and Interferon (Figure 5D). Furthermore, the effect of additional inflammatory cells (e.g., macrophages) on EV production by CAFs was tested. In contrast to the Simvastatin treatment, the production of EVs by CAFs was dramatically increased (7.79 × 10^8^ vs. 4.02 × 10^8^ in control) when CAFs were treated with EV-depleted conditioned media collected from activated (stimulated by LPS) macrophages (Figure S2B). 

## 3. Discussion

Mesothelioma is a fatal disease and the role of chronic inflammation in MPM development is well documented [1,9]. During the long latency period of this disease, mesothelial cells remain exposed to paracrine signalling from inflammatory cells recruited to the fibre-retaining areas of the mesothelium. It is not clear how different cell types contribute to the malignant transformation of mesothelial cells. In an inflammatory environment, fibroblasts assume an “activated” phenotype due to their stimulation with growth factors or cytokines [39]. The abundance of activated fibroblasts in inflamed tissue emphasises their importance in cell-to-cell communication, especially if the inflammation persists, as in the case of decades-long chronic inflammation preceding mesothelioma. CAFs comprise the majority of cell types within the MPM microenvironment, where they support tumour growth and contribute to chemoresistance [40]. A search for new therapies targeting the microenvironment requires a better understanding of CAF-specific contribution to fibre-induced carcinogenesis.

Here, we identified that CAFs promote a pro-oncogenic phenotype in normal untransformed mesothelial cells. The extensive transfer of biological material by EVs released from CAFs leads to the activation of pro-oncogenic signalling pathways and enhanced mesothelial cell proliferation and motility. Notably, the aberrant signalling induced by CAF-EVs was ameliorated by treatment with Simvastatin. 

The activated status of primary CAFs was consistent with a pattern of pro-oncogenic signalling pathways induced in MPM patients. VEGF and HGF, known key players in MPM carcinogenesis [41], were highly expressed in CAFs, suggesting CAF’s contribution to tumorigenesis. FAP, also found abundant in CAFs, was recently shown to mediate immunosuppression in a transplanted murine tumour model [42], while the release of the DAMP, high-mobility group protein B1 (HMGB1), plays a key role in sustained inflammatory signalling [43]. Cytoplasmic mRNA translocation [8] and release of HMGB1 were previously ascribed to mesothelial cells within mesothelioma tissue [44], and the role of CAFs in releasing DAMPs has so far only been reported in the context of autophagy in breast cancer [45]. The contribution of CAF-EVs to HMGB1 release and stimulation of pro-oncogenic signalling pathways reinforces the need for targeting the stroma as a therapeutic strategy. The EV cargo transferred from CAFs to normal mesothelial cells exerted a plethora of changes in the recipient cells, with marked stimulation of signalling pathways induced by activated but not normal fibroblasts evident after EV treatment. Furthermore, functional readouts demonstrated that normal mesothelial cells acquired features characteristic of pre-neoplastic status. Increased proliferation was abolished by inhibition of MAPK or Src, but not STAT3 signalling. Although STAT3 plays an important role in oncogenesis-associated proliferation, this transcription factor, if compromised, can be compensated by others, e.g., c-Myc and Pim downstream of ERK1/2 [46]. It has been shown that phosphorylation of S6 ribosomal protein correlates with an increase in translation of mRNA transcripts that encode proteins involved in cell cycle progression, as well as ribosomal proteins and elongation factors necessary for translation [47], all of which were shown to be upregulated in MPM patient samples [8]. Thus, CAFs-EV-induced increases in phospho-S6 is consistent with the increased translation in normal mesothelial cells. CAF-derived EV induction of the motile state of normal mesothelial cells was Src- and Src partner in integrin signalling FAK [48]-dependent. 

Statins are pluripotent drugs exhibiting multiple non-lipid-lowering actions. Their ability to modulate the inactivated Hippo pathway in mesothelioma, as well as inhibit AKT/mTOR, ERK and JAK/STAT3 pathways in renal cancer cells in vitro [32,49] supported Meta-Analyses based studies showing a beneficial effect of statins for both overall survival and cancer-specific survival [50,51]. Treatment with Simvastatin led to a change in the protein content in EVs released by primary CAFs from three mesothelioma patients. Over 600 proteins were identified in CAF-EVs cargo and treatment with Simvastatin eliminated/reduced/increased the presence of over 400 of those. Further analysis of the proteins whose concentration in EVs was affected by Simvastatin treatment revealed their involvement in cancer-relevant pathways. Importantly, ~50 of these proteins were not actively translated by primary normal mesothelial cells as determined by further analysis of transcriptional and proteomics data. Furthermore, the proteins in CAF-EVs cargo affected by Simvastatin displayed a strong functional link (*p* < 0.001) with those proteins that showed a low level of translation in normal mesothelial cells. Thus, upon the uptake of these proteins by normal mesothelial cells, the cellular make-up and function of the latter would change markedly, which is consistent with kinome profiling of LP-9 cells incubated with EVs from Simvastatin-treated and control CAFs.

Here, we have shown a new aspect of cellular crosstalk between primary CAFs from MPM and mesothelial cells using EVs release and uptake, resulting in the acquisition of new pro-oncogenic features by normal mesothelial cells. Treatment with Simvastatin ameliorated the pro-oncogenic effects of CAF- EVs by mechanisms involving a reduction in EV numbers and a change in EV cargo. Collectively, these data determine the significance of CAF-derived EVs in mesothelioma development and progression and suggest new targets for cancer therapy. More research will be necessary to further uncover the complex crosstalk between activated fibroblasts and other cells, not only in the tumour microenvironment but in the inflammatory microenvironment during the latency period of mesothelioma development.

## 4. Materials and Methods

### 4.1. Collection of Clinical Specimens

All patients underwent surgery for radical decortication. The resected mesothelioma specimens were collected with patient consent. Solid tumours were immediately immersed in ice-cold RPMI-1640 supplemented with penicillin (100 U/mL), streptomycin (100 μg/mL), and 10% FBS. Samples were transported to the laboratory for primary cell culture within one hour of collection. Informed consent was obtained from all subjects with Ethical Committee Approval (LREC 08/H0406/226).

### 4.2. Primary Carcinoma-Associated Fibroblast (CAF) Isolation and Treatment

CAFs were isolated from freshly resected tumours (*n* = 8 mesothelioma patients). Tumour tissue was mechanically dissociated under sterile conditions and transferred into serum-rich dissection media (RPMI-1640 media supplemented with 10% FBS); cell release was encouraged by mixing the tissue suspension. Cells were collected by centrifugation at 300× *g* followed by red blood cell lysis. Cells were seeded in gelatin-coated flasks at 5 × 10^4^/cm^2^ and maintained in RPMI-1640 growth media supplemented with L-glutamine (2 mM), penicillin (100 U/mL), streptomycin (100 μg/mL), hEGF (20 ng/mL), hydrocortisone (1 µg/mL), heparin (2 μg/mL) and 2% FBS at 37 °C and 5% CO_2_. Differential trypsinisation with 0.05% and 0.1% trypsin was used to select fibroblasts and mesothelial cells, respectively. CAF cultures from at least three individual patients were used in downstream assays. CAFs were monitored regularly for their morphology and were used at early (under 10) passages. All cell culture reagents were from Life Technologies (Paisley, UK), except for hydrocortisone and heparin (Sigma-Aldrich, Dorset, UK).

### 4.3. CAF Treatment with Simvastatin

Simvastatin (Cambridge Bioscience Ltd., Cambridge, UK) was activated as described previously [52]. Briefly, to activate simvastatin provided in the form of a lactone prodrug, it was hydrolysed in ethanolic NaOH [15% (*v*/*v*) ethanol and 0.25% (*w*/*v*) NaOH] at 50 °C for 2 h. The pH was brought to 7.0 by HCl, and the final concentration of the stock solution was adjusted to 500 mM. A stock solution of the hydrolysed simvastatin was stored at 20 °C. CAFs were treated with 500 nM Simvastatin 24 h prior to EVs collection. 

### 4.4. Cell Cultures and Treatments

Adult human primary omental mesothelial cells (single or multi-donor) purchased from Cambridge Bioscience (Cambridge, UK) were maintained in culture in a growth medium (Medium 199, 10% FBS, 20 ng/mL hEGF, 100 U/mL penicillin, 100 μg/mL streptomycin) for up to three passages. Normal human untransformed mesothelial cells LP-9 were purchased from the Coriell Institute for Medical Research (Camden, NJ, USA) and maintained in RPMI-1640 growth media supplemented with L-glutamine (2 mM), penicillin (100 U/mL), streptomycin (100 μg/mL), hEGF (20 ng/mL), hydrocortisone (1 µg/mL), heparin (2 μg/mL) and 10% FBS at 37 °C and 5% CO_2_. 24 h prior to experimentation, LP-9 cells were seeded in 6-well plates at a concentration of 1.5 × 10^5^ /cm^2^. All cell culture reagents were from Life Technologies (Paisley, UK), except for hydrocortisone and heparin (Sigma-Aldrich, Dorset, UK). LP-9 treatment with the kinase inhibitors for ^35^S Met incorporation and motility assays was performed as follows: 10 µM U0126 [1,4-diamino-2,3-dicyano-1,4-bis(*o*-aminophenylmercapto) butadiene] (Calbiochem, La Jolla, CA, USA), 50 µM LY294002 [2-(4-morpholinyl)-8-phenyl-1(4*H*)-benzopyran-4-one] (Cell Signaling Technology, Buckinghamshire, UK), 50 nM PP2 [4-amino-5-(4-chlorophenyl)-7-(*t*-butyl)pyrazolo [3,4-*d*]pyrimidine] (Calbiochem, La Jolla, CA, USA), 1 µM PF-573228 (3,4-Dihydro-6-[[4-[[[3-(methylsulfonyl) phenyl]methyl]amino]-5-(trifluoromethyl)-2-pyrimidinyl]amino]-2(1*H*)-quinolinone) (Bio-Techne, Abingdon, UK) and 10 µM S3I-201 (2-hydroxy-4-[[2-[[(4-methylphenyl) sulfonyl] oxy]acetyl]amino]-benzoic acid) (Cambridge Bioscience Ltd., Cambridge, UK) for 24 h.

### 4.5. Extracellular Vesicle (EV) Purification

EVs were isolated from CAFs and NDHFs. Briefly, fibroblasts were seeded at 0.2 × 10^5^/cm^2^; when cells reached 80% confluence, the medium was changed to an EV-depleted medium, prepared by ultracentrifugation of FBS (120,000× *g*, 4 h). Conditioned medium was collected 24 h later for EV isolation by differential centrifugation as described previously [53]; briefly, centrifugation at 300× *g* for 10 min, followed by 20 min at 16,500× *g*. After each centrifugation, the supernatant was transferred into a new test tube while the generated pellets were discarded. After the 16,500× *g* spin, supernatants were filtered (0.22 µm filter units, cellulose acetate) and ultracentrifuged at 120,000× *g* for 70 min (SW40 rotor; Beckman Coulter, High Wycombe, UK). Pellets were then resuspended in RPMI and immediately used for the treatment of normal mesothelial cells. As EVs could only be isolated in limited amounts, MISEV [25] was not fully followed; instead, we prioritised the characterisation of EVs via Electron Microscopy and Nanoparticle Tracking analysis.

### 4.6. Electron Microscopy

For transmission electron microscopy (TEM), EV pellets were fixed in 2% glutaraldehyde in 0.1 M sodium cacodylate buffer (pH 7.4) at 4 °C overnight and post-fixed with 1% osmium tetroxide/1% potassium ferrocyanide for 1 h at room temperature. After fixation, EVs were stained en bloc with 5% aqueous uranyl acetate overnight at room temperature, dehydrated in a series of alcohols and embedded in Taab epoxy resin (Taab Laboratories Equipment Ltd., Aldermaston, UK). Ultrathin sections were stained with lead citrate and recorded using a Megaview 3 digital camera and iTEM software (Olympus Soft Imaging Solutions GmbH, Münster, Germany) in a Jeol 100-CXII electron microscope (Jeol UK Ltd., Welwyn Garden City, UK).

For scanning electron microscopy (SEM), cells on glass coverslips were processed to absolute alcohol, as for TEM, transferred to hexamethyldisilazane and air-dried before being sputter-coated with a 15 nm layer of gold and examined in an FEI Quanta FEG 250 electron microscope (FEI Europe, Eindhoven, The Netherlands).

### 4.7. Extracellular Vesicle Tracking Analysis

A Nanoparticle Tracking Analyser (NTA) (Malvern Instruments Ltd., Malvern, UK) with an LM14 view unit, blue laser (405 nm, 70 mW) and an sCMOS camera (Hamamatsu Photonics, Hamamatsu, Japan) was used to measure the size-distribution and concentration of EVs. Triplicate measurements under constant equipment settings were conducted as follows: camera level 14, auto-settings off, reproducibility and polydispersity high, acquisition time 90 s, <100 particles per image, screen gain 10, and threshold 10. Data analysis was performed with NTA 2.3 software (NanoSight, Amesbury, UK).

### 4.8. Western Blot Analysis

Cells were lysed for 20 min on ice in lysis buffer (1% NP-40, 20 mM Tris-HCl, pH 8.0, 137 mM NaCl, 10% glycerol, 2 mM EDTA, 1 mM sodium orthovanadate, 10 μg/μL leupeptin, and 10 μg/μL aprotinin) (Sigma-Aldrich, Dorset, UK), briefly sonicated and the resultant lysate clarified by centrifugation at 1000× *g* 10 min. Protein content was measured by Bradford assay and 20 μg protein was loaded onto 4–20% Criterion TGX gel (Bio-Rad Laboratories, Hemel Hempstead, UK). Proteins were blotted onto polyvinylidene difluoride membranes (Millipore, Watford, UK). Immunoblotting was done after blocking with 5% non-fat milk with the following primary antibodies: anti-phospho-Akt (Ser473) anti-Akt, anti-S6 Ribosomal Protein, anti-phospho-S6 Ribosomal Protein (Ser235/236), anti-STAT3, anti-phospho-STAT3 (Tyr705), anti-Src, Anti-phospho-Src (Tyr416), anti-phospho-ERK1/2 (Thr202/Tyr204), anti- ERK1/2, anti-HMGB1, anti-GAPDH (all Cell Signalling Technology, Buckinghamshire, UK). Results were quantified using densitometry and Image Quant TL software. Statistical significance was estimated using a two-tailed Student’s *t*-test, * *p* < 0.05.

### 4.9. Immunofluorescence

After treatment, cells were fixed with 4% paraformaldehyde at room temperature for 20 min and permeabilised with 0.2% Triton X-100 (Sigma-Aldrich, Dorset, UK) in PBS for 5 min. Cells were then incubated with primary antibody 1:500 anti-Vimentin; 1:500 anti-HMGB1; 1:500 anti-VEGF 1:500 (Cell Signalling Technology, Buckinghamshire, UK); 1:500 anti-FAP (R&D Systems, Oxford, UK); 1:500 anti-HGF and 1:250 anti-STAT3 (Abcam, Cambridge, UK) overnight at 4 °C. Cells were washed three times with PBS, incubated with secondary antibody for 1 h, counterstained with 300 nM 4-6-diamidino-2-phenylindole for 10 min and mounted for confocal microscopy. Secondary antibodies alone were used as specificity controls and uniformly resulted in very low background levels of fluorescence. For EV uptake, exosomes were labelled with PKH26 (Sigma-Aldrich, Dorset, UK) according to the manufacturer’s protocol. Briefly, exosome pellets were resuspended in 1 mL Diluent C. Separately, 1 mL Diluent C was mixed with 4 μL PKH26. The exosome suspension was mixed with the stain solution and incubated for four minutes; staining was stopped by the addition of 10 mL of culture medium followed by ultracentrifugation at 100,000× *g* for 70 min. Labelled exosomes were resuspended in culture media and added to LP-9 cells. To visualise the internalisation of EV, LP-9 cells were fixed after 0 h, 1 h, and 10 h after treatment and analysed by confocal microscopy.

### 4.10. Kinome Profiling

Kinome profiling was performed using a Phospho-kinase Array Kit (64 Kinases) according to the manufacturer’s protocol (R&D Systems, Oxford, UK). In brief, LP-9 cells untreated or treated with EVs were lysed. Lysates, cleared by centrifugation, were loaded onto the provided membranes pre-coated with capture antibodies, and the presence of bound phospho-proteins was determined by western blotting with a mixture of detection antibodies. The signal intensities for kinase phosphorylation were determined in duplicate by densitometry and normalised to the positive control provided.

### 4.11. Motility Assay

LP-9 cells were plated in 6-cm plates at 2 × 10^5^ cells/cm^2^ and treated with equal amounts of EVs with or without kinase inhibitors. Cell motility was analysed by time-lapse video microscopy (16 h, 15 min acquisition interval at 37 °C and 5% CO_2_) using an Axiovert 200 M microscope and MetaMorph^®^ NX 2.5 software (San Jose, CA, USA). Manual cell tracking analysis was performed with ImageJ as described previously [54] in three independent experiments. Error bars show mean ± SD. Statistical significance was estimated using a two-tailed Student’s *t*-test, * *p* < 0.05.

### 4.12. Met ^35^S Incorporation

LP-9 cells were plated in 6-cm plates at 5 × 10^5^ cells/cm^2^ and treated with equal amounts of EVs with or without kinase inhibitors. After 24 h treatment, cells were washed with PBS, and the medium was replaced with methionine and cysteine-free MEM containing 10% dialysed FBS. Thirty minutes later, 250 μCi/mL [^35^S]-methionine/cysteine. After a further 20 min incubation, cells were placed on ice and washed twice in ice-cold PBS and then lysed in 600 μL of lysis buffer (10 mM Tris at pH 7.5, 50 mM NaCl, 0.5% NP-40, 0.5% deoxycholate, 0.5% SDS). Proteins were precipitated onto Whatman filter paper (Sigma-Aldrich, Dorset, UK) by the addition of trichloroacetic acid to 12.5% and washed with 70% IMS and then acetone. Radioactivity was measured by scintillation counting; scintillation was read from dried filter paper in triplicate for each experimental condition. Total protein content was determined by Bradford assay (Sigma-Aldrich, Dorset, UK) and ^35^S-methionine incorporation standardised against protein content. Error bars show mean ± SD from three independent experiments. Statistical significance was estimated using a two-tailed Student’s *t*-test, * *p* < 0.05.

### 4.13. Mass Spectrometry

After destaining according to the manufacturer’s instructions, gels were serially sectioned, digested with trypsin and peptides extracted as described [55]. Dried tryptic peptides were resuspended in injection solvent (5% formic acid (aq) and 10% acetonitrile (aq) (9:1)) and spiked with 40 fmol/µL MassPREP standards (Waters Corporation, Manchester, UK), using yeast alcohol dehydrogenase (ADH1, accession: P00330) and bovine serum albumin (ALBU, accession: P02769). Nanoscale UPLC separation of tryptic peptides was performed on a nanoAcquity UPLC system (Waters) equipped with a 25 cm × 75 µm I.D., 1.7 µm, BEH130 C18 analytical reverse phase column. Samples (2–4 µL injections) were separated using 50 min, 3% to 40% acetonitrile gradients at 0.3 µL/min. Mass spectrometric analysis of eluted peptides was carried out using a Waters Synapt G2-S HDMS mass spectrometer, equipped with T-Wave-IMS and performed in data-independent acquisition (DIA) and ion mobility modes (HDMS^E^), with a travelling wave velocity of 650 m/s. Peptide fragmentation was performed by stepping between 4 eV (low energy) and 20–50 eV (collision-induced dissociation) voltages. Low energy and CID data were acquired with a 1-s cycle scan time and 50–2000 *m/z* mass range. LC-MS data were processed and searched using Waters ProteinLynx Global SERVER version 3.0 (PLGS, Waters) and identified using the UniProt Human reviewed database (UniProtKB release 2014_11, 20,265 entries). Raw data files were also analysed using PLGS version 3 and IsoQuant. These data were used for the “top 3” absolute quantification of proteins. For database searching in PLGS, peptide mass tolerance and fragment mass tolerance were set to auto, with one missed cleavage and variable modification for methionine oxidation. A false discovery rate (FDR) of 1% was used for PLGS and IsoQuant analysis, an FDR of 0.1% was used with only the three most abundant unmodified peptides used for quantification. The data were also analysed using Scaffold version 3.3.1 software (Proteome Software Inc., Portland, OR, USA) as described [55].

### 4.14. RNA Microarrays

Total RNA from primary normal mesothelial cells and from primary mesothelioma cells isolated from 4 patients was extracted by TRIzol^TM^ (Fisher Scientific, Loughborough, UK) and then used for labelling and hybridisation. Hybridisation to 60 K whole human genome microarray gene expression chips was conducted following the manufacturer’s protocol (Agilent Technologies, Berkshire, UK). Briefly, total RNA from primary normal mesothelial cells and primary MPM cells were used for labelling and hybridisation. RNA samples were Cy3-labelled using Agilent Low Input Quick Amp 1-colour Labelling Kit (Agilent Technologies, Berkshire, UK). The level of dye incorporation was evaluated using a spectrophotometer (Nanodrop ND1000, LabTech, East Sussex, UK). Labelled RNA was then fragmented in the appropriate buffer (Agilent Technologies, Berkshire, UK) for 30 min at 60 °C before dilution (*v*/*v*) in the hybridisation buffer. Hybridisation to 60 K high-density oligonucleotide microarray slides was performed in a microarray hybridisation oven (Agilent Technologies, Berkshire, UK) overnight at 65 °C. Following hybridisation, the slides were rinsed in gene expression wash buffers 1 and 2 and immediately scanned using a DNA Microarray Scanner (Model G2505C, Agilent Technologies, Berkshire, UK).

### 4.15. Analysis of Microarray Data

The raw data was uploaded into Agilent’s GeneSpring Software, normalised and fold changes calculated. For each MPM cell line, the probes with an absolute 2-fold-change in mRNA expression between primary normal and malignant cell lines were included in subsequent analyses. Dataset for mRNA array, the “Gene changes” have been deposited in the EBI ArrayExpress/BioStudies database www.ebi.ac.uk/biostudies/arrayexpress/studies (accessed on 30 May 2022) under ID code: E-MTAB-6351 (http://www.ebi.ac.uk/biostudies/arrayexpress/studies/E-MTAB-6351 (accessed on 30 May 2022)).

The network-based statistical framework was used to demonstrate a strong functional dependency between CAF-EV proteins affected by Simvastatin (further referred to as list **a**) and genes significantly downregulated in normal mesothelial cells compared to malignant cells (further referred to as list **b**) employing available knowledge of gene signalling pathways and networks. For each protein (denoted as **A**) from list **a,** the following procedure was repeated. First, the distance from protein **A** to all genes from list ***b*** was computed using a reference gene network. The distance was defined as a minimal number of steps required to get from one gene to another using the edges of the network. Second, the connectivity score ***S_Ab_*** (between protein ***A*** and list ***b***) based on the number of genes from list ***b*** with distances 1 and 2 to protein ***A*** was defined. To find the statistical significance of the connectivity score, the Monte Carlo procedure was used [56]. A gene list “***r***” equivalent in size to the list ***b*** was sampled randomly, followed by a computation of the connectivity score ***S_Ar_*** (between protein ***A*** and the list “***r***”). The procedure was repeated N times (up to N = 10,000 if required) to find out the empirical distribution {***S_Ar_***}***_k_*** (***k*** = 1, 2, …, N) of the connectivity score between protein ***A*** and a random gene list (equivalent in size to the input list ***b***). The estimate of significance (*p*-value) for the score ***S_Ab_*** was computed as *p* = (*v* + 1)/N, where *v* is the number of times, the ***S_Ab_*** was less or equal to the scores from the ***S_rA_*** distribution. The procedure is repeated for each protein “***A***” from the input list **a**; therefore, a number of hypotheses (equal to the number of genes in list ***A***) are tested. Standard FDR procedure [57] was applied to adjust *p*-values for multiple testing.

## Data Availability

Publicly available datasets were analyzed in this study. This data can be found here: http://www.ebi.ac.uk/biostudies/arrayexpress/studies/E-MTAB-6351, accessed on 30 May 2022.

## Supplementary Materials

The following supporting information can be downloaded at: https://www.mdpi.com/article/10.3390/ijms232012469/s1.
