# Supplementary material for: Extracellular Vesicles Isolated from Malignant Mesothelioma Cancer-Associated Fibroblasts Induce Pro-Oncogenic Changes in Healthy Mesothelial Cells
Source: Int J Mol Sci. 2022 Oct 18;23(20):12469. doi: 10.3390/ijms232012469 (PMC9604431; doi:10.3390/ijms232012469)
Supplement: Supplementary file 1 [file ijms-23-12469-s001.zip › ijms-1775087-supplementary.pdf]

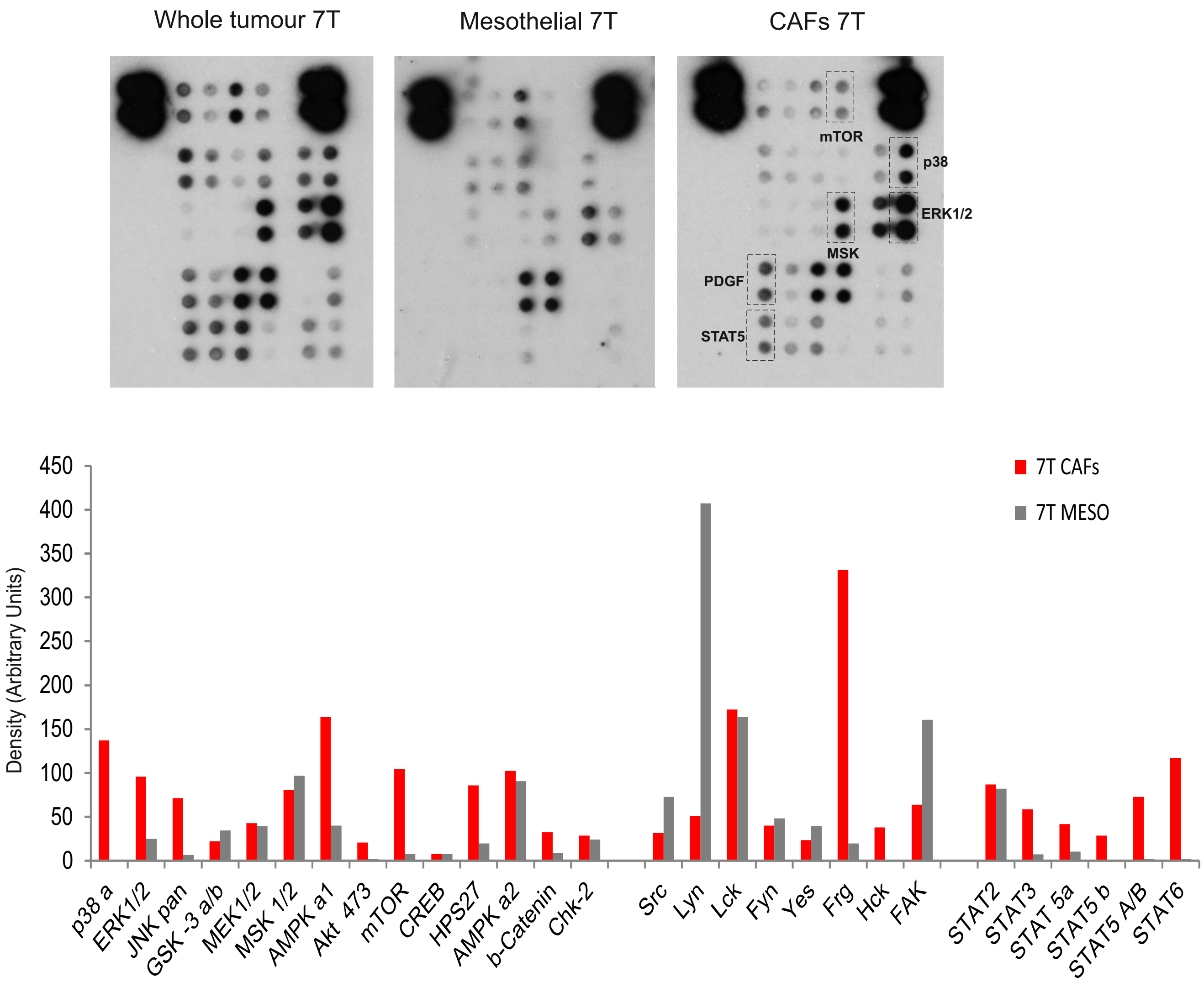

Supplemental Figure S1. Differential contribution of tumour cells and CAFs to activation of signalling pathways in the whole tumour. **A.** The image shows the immunoblots of kinases in whole mesothelioma tumour 7T tissue (left), in primary mesothelial (mesothelioma) cells derived from tumour 7T (middle) and in CAFs derived from tumour 7T (right). Signals from the most differentially activated kinases are boxed. **B.** Kinase phosphorylation status in 7T CAFs and 7T Mesothelioma cells quantified by densitometry.

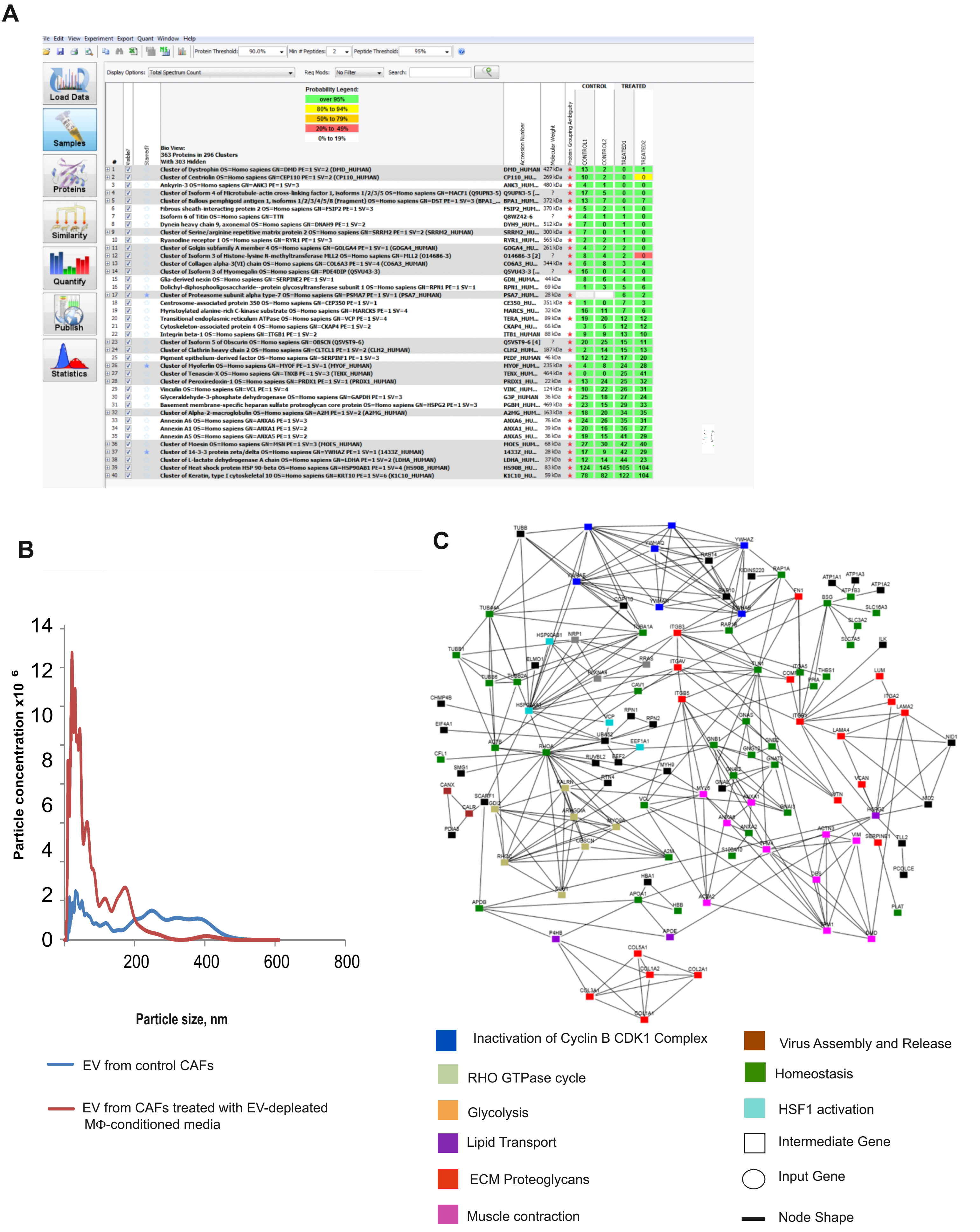

Supplemental Figure S2. Changes in CAF-EVs protein content by CAF treatment with Simvastatin. **A.** An extract from the Mass Spectrometry results showing the EV proteins with a changed concentration (reduced or increased) after treatment of CAFs with Simvastatin. **B.** Conditioned medium from activated macrophages increases production of EVs by CAFs. EV-depleted conditioned media (10 ml) from THP-1 cells stimulated with 10 μg/ml LPS was added to cultured CAFs 24 h prior to EV collection. CAF-EVs were quantified by nanoparticle tracking analysis (NTA). **C.** Differentially present proteins in EVs from Simvastatin-treated and untreated CAFs form a network of the pathways related to cell cycle, metabolism, growth factors, integrins, homeostasis and lipid transport.

Table S1. Top down-regulated genes (Database REACTOME)

#	Gene	P-value FDR	Network Model CytoScape	
1	CTNB1_HUMAN	< 0.000961538 (0.0001)	view	
2	PSA7_HUMAN	< 0.000961538 (0.0001)	view	
3	RAN_HUMAN	~ 0.000961538 (0.0001)	view	
4	RL40_HUMAN	< 0.000961538 (0.0001)	view	
5	U520_HUMAN	< 0.000961538 (0.0001)	view	
6	HSP7C_HUMAN	< 0.000961538 (0.0001)	view	
7	TERA_HUMAN	~ 0.000961538 (0.0001)	view	
8	GELS_HUMAN	< 0.000961538 (0.0001)	view	
9	RS3_HUMAN	< 0.000961538 (0.0001)	view	
10	HSPB1_HUMAN	< 0.000961538 (0.0001)	view	
11	RPN1_HUMAN	< 0.000961538 (0.0001)	view	
12	IF2B_HUMAN	< 0.000961538 (0.0001)	view	
13	EIF3I_HUMAN	< 0.000961538 (0.0001)	view	
14	IF4A2_HUMAN	< 0.000961538 (0.0001)	view	
15	CDC26_HUMAN	< 0.000961538 (0.0001)	view	
16	RLA2_HUMAN	< 0.000961538 (0.0001)	view	
17	HSP71_HUMAN	< 0.000961538 (0.0001)	view	
18	RPN2_HUMAN	< 0.000961538 (0.0001)	view	
19	VIME_HUMAN	< 0.000961538 (0.0001)	view	
20	CHM4B_HUMAN	< 0.000961538 (0.0001)	view	
21	ATPB_HUMAN	< 0.000961538 (0.0001)	view	
22	EF1A1_HUMAN	< 0.000961538 (0.0001)	view	
23	PTRF_HUMAN	< 0.000961538 (0.0001)	view	
24	PRIC1_HUMAN	< 0.000961538 (0.0001)	view	
25	IF4A1_HUMAN	< 0.000961538 (0.0001)	view	

Table S2. Top down-regulated genes (Data base INTACT)

#	Gene	P-value FDR	Network Model CytoScape	
1	THIO_HUMAN	< 0.000213904 (0.0001)	view	
2	PRDX6_HUMAN	< 0.000213904 (0.0001)	view	
3	PGBM_HUMAN	< 0.000213904 (0.0001)	view	
4	K2C1B_HUMAN	< 0.000213904 (0.0001)	view	
5	GBB1_HUMAN	< 0.000213904 (0.0001)	view	
6	K1C16_HUMAN	< 0.000213904 (0.0001)	view	
7	S10AA_HUMAN	< 0.000213904 (0.0001)	view	
8	PSA7_HUMAN	< 0.000213904 (0.0001)	view	
9	H90B2_HUMAN	< 0.000213904 (0.0001)	view	
10	HSP7C_HUMAN	~ 0.000213904 (0.0001)	view	
11	HS902_HUMAN	< 0.000213904 (0.0001)	view	
12	AHNK_HUMAN	< 0.000213904 (0.0001)	view	
13	TMEDA_HUMAN	< 0.000213904 (0.0001)	view	
14	K2C1_HUMAN	< 0.000213904 (0.0001)	view	
15	ALDOA_HUMAN	< 0.000213904 (0.0001)	view	
16	ITB1_HUMAN	< 0.000213904 (0.0001)	view	
17	RS3_HUMAN	< 0.000213904 (0.0001)	view	
18	HSPB1_HUMAN	< 0.000213904 (0.0001)	view	
19	GBG12_HUMAN	< 0.000213904 (0.0001)	view	
20	PPIB_HUMAN	< 0.000213904 (0.0001)	view	
21	RHOC_HUMAN	< 0.000213904 (0.0001)	view	
22	TSP1_HUMAN	< 0.000213904 (0.0001)	view	
23	CALR_HUMAN	< 0.000213904 (0.0001)	view	
24	FKB1A_HUMAN	< 0.000213904 (0.0001)	view	
25	RPN1_HUMAN	< 0.000213904 (0.0001)	view	
26	RAC2_HUMAN	< 0.000213904 (0.0001)	view	
27	EIF3I_HUMAN	< 0.000213904 (0.0001)	view	
28	GDIB_HUMAN	< 0.000213904 (0.0001)	view	
29	RAB1B_HUMAN	< 0.000213904 (0.0001)	view	
30	APOE_HUMAN	< 0.000213904 (0.0001)	view	
